# Dictyophora Polysaccharide Attenuates As-Mediated PINK1/Parkin Pathway-Induced Mitophagy in L-02 Cell through Scavenging ROS

**DOI:** 10.3390/molecules27092806

**Published:** 2022-04-28

**Authors:** Ting Hu, Ju Lu, Changyan Wu, Tianxiao Duan, Peng Luo

**Affiliations:** 1 School of Public Health, Guizhou Medical University, Guiyang 550025, China; hutinggmc@126.com (T.H.); luju3621luju@163.com (J.L.); wcy9409@163.com (C.W.); txduan2020@hotmail.com (T.D.); 2 Key Laboratory of Environmental Pollution Monitoring Control Ministry of Education, Guizhou Medical University, Guiyang 550025, China; 3 Guizhou Engineering Research Center of Food Nutrition and Health, Guiyang 550025, China; 4 State Key Laboratory of Functions and Applications of Medicinal Plants, Guizhou Medical University, Guiyang 550014, China

**Keywords:** Dictyophora polysaccharide, As, PINK1/Parkin, ROS

## Abstract

Arsenic (As) is common in the human living environment and a certain amount of exposure to As can lead to liver damage; this toxic effect has been proved to be closely related to intracellular PINK1/Parkin pathway-mediated mitophagy. Dictyophora is an edible fungus that extracts polysaccharides with antioxidant and hepatoprotective effects. In the present study, we demonstrated that As induced the onset of mitophagy in hepatocytes by stimulating cellular production of ROS to activate PINK1/Parkin, and the extent of damage increased with increased As-induced toxicity. Dictyophora polysaccharide (DIP) has the ability to scavenge intracellular ROS, which can inhibit oxidative stress injury and inhibit the PINK/Parkin pathway through its receptors or efficacious proteins, thus preventing mitochondrial autophagy and alleviating the hepatotoxicity of As. In conclusion, our results indicate that DIP can reduce As-induced PINK1/Parkin pathway-mediated hepatic mitophagy through scavenging ROS and exert hepatoprotective effects, providing experimental data and theoretical basis for the development of medicinal value of Dictyophora as a dual-use food and medicinal fungus.

## 1. Introduction

Arsenic (As) is a basic element for animals and is predominantly found in rocks, soils, and natural water [1]. Many studies have shown that oxidative stress, cell proliferation, and induction of cell death have been reported followed by arsenic exposure [2,3,4]. Long-term arsenic exposure can cause damage to multiple organs in the body, with the liver being one of the main target organs [5]. Normal activities of the liver are dependent on mitophagy in many liver diseases [6]. Mitophagy is a selective autophagy that specifically degrades dysfunctional or superfluous mitochondria and is one of the most important mitochondrial quality control mechanisms. This is essential for maintaining cellular homeostasis [7,8]. Our previous studies have found that arsenic accumulation in liver cells caused some amount of damage, and this toxic effect was mediated by the PINK1/Parkin signaling pathway [9], which is well-recognized for being one of the key pathways that mediated mitophagy [10].

Dictyophora is one of the most precious and important plant resource in China and is one of the highly valued mushroom in the world [11]. It is also regarded as the “queen of the mushrooms” [12] and has food and medicinal effects, i.e., antifatigue, hypoglycemic, immunomodulatory, antiaging, and hepato-protective effects [13]. Recently, our studies in rats exposed to sodium arsenite showed that Polysaccharides extracted from Dictyophora (DIP) could alleviate liver injury. It suggested that DIP has medicinal value in protecting liver injury [14]. However, the underlying mechanisms are still unclear. Whether the hepatoprotective effect of DIP is related to mitophagy needs to be further clarified. In our study, L-02 hepatocyte was treated with sodium arsenite to explore the effect of DIP in sodium arsenite-induced liver injury and the mediating role of mitophagy. The results of this study will lay a foundation for the development of nutrition and medicinal value of Dictyophora chinensis.

## 2. Materials and Methods

### 2.1. Materials

Normal human liver cell line L-02 (Cell Bank of Chinese Academy of Sciences); Dictyophora was picked in Zhijin County (Bijie, Guizhou, China); Sodium arsenite, N-acetylcysteine (Sigma, New York, NY, USA); RPMI1640 medium, fetal bovine serum, pancreatin, (Gibco, Waltham, MA, USA); CCK-8 cell proliferation toxicity detection kit (Shanghai Dongren Chemical Technology Co., Ltd., Shanghai, China); reactive oxygen detection kit, BAC protein concentration determination kit (Beyotime, Shanghai, China); rapid reverse transcription kit, qPCR kit (Yishan Biotechnology, Shanghai, China); β-tublin primary antibody (Aibixin, Shanghai, China), LC3 II primary antibody, p62 primary antibody, PINK1, Parkin primary antibody (CST, Kansas City, MO, USA); CO_2_ incubator (INC108med, Memmert, Schwabach, Germany); high-speed refrigerated centrifuge (3K15, Sigma, USA); microplate reader (Multiskan FC, Thermo Fisher, Waltham, MA, USA); flow cytometer (NovoCyte, Agilent, Santa Clara, CA, USA); timing voltage stabilized steady flow electrophoresis instrument (PowerPac-Basic, Bio-Rad, Hercules, CA, USA); electrophoresis and transfer tank (MiniPROTEAN Tetra, Bio-Rad, Hercules, CA, USA); gel imaging system (Chemic+, Bio-Rad, Hercules, CA, USA).

### 2.2. Cell Culture

L-02 cells were cultured in RPMI-1640 medium containing 10% fetal bovine serum and 1% penicillin-streptomycin mixture and kept in a humidified incubator with 95% air and 5% CO_2_ at 37 °C. Cells in the normal control group were cultured normally and the treatment group was pretreated with NaAsO_2_ or DIP for 4 h and then exposed to 10 μM NaAsO_2_. DIP was extracted from the fruit body of Dictyophora purchased by us, and the specific extraction method was the same as in our study [15]. We have confirmed that DIP is mainly a polysaccharide composed of D-glucose, and the rest include D-mannose, L-rhamnose, D-galactose, D-xylose, L-fucose, etc. [16]. All experiments were performed 24 h after cell inoculation.

### 2.3. Cell Viability Assay

The proliferation effect of NaAsO_2_ or DIP on L-02 cells was detected using an CCK-8 kit. According to the manufacturer’s instruction, cells were cultured in 96-well plates at approximately 1 × 10^4^ cells per well. When the cells were adherent to the bottom of the cell, the cells were treated with different concentrations of NaAsO_2_ for 24 h or pretreated with different concentrations of DIP for 4 h and then exposed to 10 μM NaAsO_2_ for 24 h. Then, CCK-8 was added and incubated for 2–4 h at 37 °C. The absorbance at 490 nm was measured using a microplate reader. The data were calculated from the mean of six replicates; each experiment was conducted in triplicate.

### 2.4. Detection of ROS

L-02 cells were treated with NaAsO_2_ (10 μM, 24 h), DIP (80 μg/mL, 4 h, then exposed to 10 μM NaAsO_2_ for 24 h), and NAC (5 mM, 1 h, then exposed to 10 μM NaAsO_2_ for 24 h). One set of L-02 cells without any treatment was kept as normal control. Furthermore, 1 mL of DCFH-DA with a final concentration of 10 μmol/L was added to each well. The solution was then incubated for 20 min at 37 °C, the serum-free culture solution was washed three times to wash out the excess of DCFH-DA, the cells were collected, and the solution was then centrifuged at 4 °C at 1000 g/min for 5 min. The cells were fixed for analysis in a flow cytometer.

### 2.5. Observe by Transmission Electron Microscopy

After cells were treated as described above, the cells were trypsinized and collected, washed twice with pre-cooled PBS, and fixed in 2.5% glutaraldehyde at 4 °C for 24 h. Then, they were washed three times with 0.1 mol/L PBS for 15 min each time, fixed with 1% osmium acid at 4 °C for 2 h, washed three times with 0.1 mol/L PBS for 15 min each time, dehydrated in gradient (50%, 70%, 90%, 100%) acetone solution for 30 min, put in anhydrous acetone with a ratio of 1:1, infiltrated with resin for 12 h, and then put the pure resin at medium penetration for 12 h. The infiltrated samples were polymerized and embedded in the embedding box at 37 °C for 12 h, 45 °C for 12 h, and 60 °C for 12 h. After ultrathin sectioning and staining, the ultrastructure of cells was observed under a transmission electron microscopy.

### 2.6. RT-qPCR

Total RNA was isolated from cells using the acid guanidinium thiocyanate-ephenol-echloroform method. A Qubit Reagent RNA concentration kit was used to determine the concentration. An appropriate amount of dissolved RNA was taken out and then reverse transcribed into cDNA. The reverse transcription product was mixed with SYBR Green, primers, and ddH_2_O according to the instructions and the reaction program was set up, and the expression of PINK1 and Parkin genes was tested on a computer. The primers were as follows: PINK1 forward, 5′-AGTCCATTGGTAAGGGCTGC-3′, and reverse, 5′-AAATCTGCGATCACCAGCCA-3′; Parkin forward, 5′-TAGCTTTGCACCTGATCGCA-3′, and reverse, 5′-GCGGCTCTTTCATCGACTCT-3′; β-actin forward, 5′-CCTGGCACCCAGCACAAT-3′, and reverse, 5′-GCCGATCCACACGGAGTA-3′; β-actin was used as a control. Relative expression levels were measured using the ∆∆Ct method.

### 2.7. Western Blot

After L-02 cells were grouped and processed as described above, an appropriate amount of lysis solution was added, and the cells were collected by a cell scraper. After the cells were fully lysed on ice, the cells were centrifuged at 12,000× *g* for 15 min. After 12% SDS-PAGE electrophoresis and transfer membrane, 5% skimmed milk powder was blocked at room temperature for 2 h, and then the LC3 II/І, p62, PINK1, Parkin, and β-tubulin antibodies were 1:1000, 1:3000, and 1:1500, respectively. The cells were incubated overnight at 1:500 and 1:3000, then incubated with horseradish peroxidase (HRP)-labeled secondary antibody (1:10,000) at room temperature for 60 min, and developed with ECL luminescent solution. Image J software was used to analyze the gray value of the protein bands.

### 2.8. Statistical Analysis

SPSS20.0 statistical software was used to analyze the data; one-way analysis of variance was used to compare the differences between groups. An LSD test was used when the variance was uniform, and Dunnett’s T3 test was used when the variance was uneven. *p* < 0.05 was considered statistically significant.

## 3. Results

### 3.1. Effect of As on the Viability of L-02 Cells

**Figure 1:**
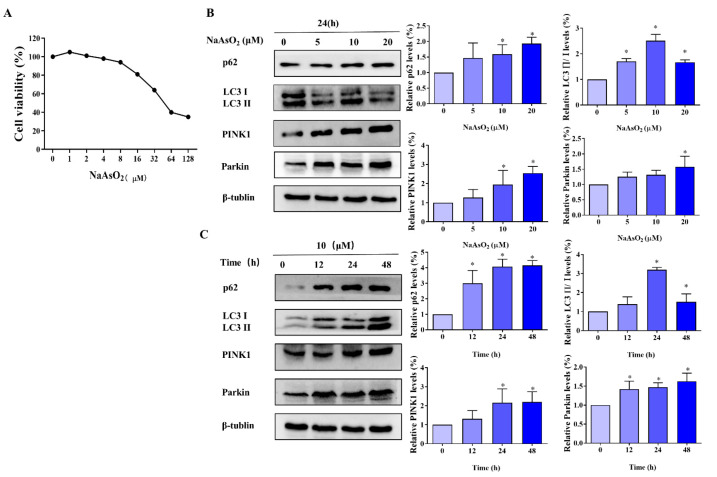
As induces mitophagy in L-02 cells. (**A**) CCK8 assay was used to explore the most suitable exposure time and concentration for this experiment. (**B**) Western blotting was used to detect the effect of different concentrations of NaAsO_2_ on autophagy-related proteins under different exposure times. (**C**) Western blotting was used to detect the effects of different exposure times on autophagy-related proteins under different concentrations of NaAsO_2_. * *p* < 0.05 compared with the control group.

L-02 cells at around 70% confluences were cultured in different concentrations (0, 1, 2, 4, 8, 16, 32, 64, and 128 μM) of sodium arsenite (NaAsO_2_) for 24 h. The CCK-8 kit was used to detect the effect of NaAsO_2_ on the viability of the L-02 cells. As shown in Figure 1A, the viability of the L-02 cells decreased in a dose-dependent manner (*p* < 0.05). It was determined that the IC_50_ of NaAsO_2_ in L-02 cells was 39.70 ± 3.59 μM; thus, we chose 1/2 IC_50_ (20 μM), 1/4 IC_50_ (10 μM), and 1/8 IC_50_ (5 μM) treated L-02 cells for 24 h as the treatment condition in the subsequent experiments.

### 3.2. As-Induces Mitophagy in L-02 Cells

To determine the occurrence of mitophagy in L-02 cells treated with NaAsO_2_, L-02 cells were treated with 5 μM, 10 μM, and 20 μM NaAsO_2_ for 12 h, 24 h, and 48 h, and the mitophagy-related proteins PINK1, Parkin, p62 and LC3 II/І were examined. The results showed that with increasing NaAsO_2_ exposure time and concentration, the overall expression of PINK1, Parkin, p62, and LC3 II/І increased (*p* < 0.05, Figure 1B,C). After L-02 cells were exposed to 10 μM and 20 μM of NaAsO_2_, the difference was statistically significant (*p* < 0.05). After L-02 cells were exposed for 24 h and 48 h, the difference was statistically significant (*p* < 0.05). Based on these results, we chose a NaAsO_2_ exposure time at 24 h and a concentration at 10 μM as the conditions for the subsequent experiments.

### 3.3. DIP Inhibited NaAsO_2_-Induced ROS and Mitophagy in L-02 Cells

**Figure 2:**
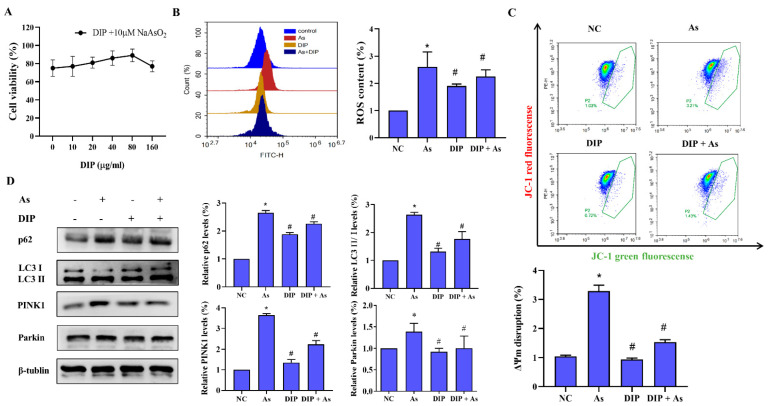
DIP inhibited NaAsO2-induced mitophagy in L-02 cells. (**A**) CCK8 assay was used to determine the concentration of DIP with the best effect. (**B**) DCFH-DA fluorescent probe was used to detect intracellular ROS. (**C**) JC-1 mitochondrial membrane potential detection kit was used to detect mitochondrial membrane potential of cells. (**D**) Western blotting was used to detect the effect of DIP on autophagy-related proteins. * *p* < 0.05 compared with the control group. # *p* < 0.05 compared with the As group.

To explore the effect of DIP against arsenic-induced mitophagy, L-02 cells were pretreated with 10 μg/mL, 20 μg/mL, 40 μg/mL, 80 μg/mL, and 160 μg/mL DIP for 4 h and then exposed to 10 μM NaAsO_2_ for 24 h. The altered activity of L-02 cells was detected by CCK8 assay. It was clear that pretreatment with 80 μg/mL DIP significantly enhanced the tolerance of L-02 cells to NaAsO_2_. Subsequent experiments chose 80 μg/mL as the DIP intervention concentration (Figure 2A). DIP has been reported to have antioxidant function by numerous studies [17,18]. Therefore, the DCFH-DA fluorescent probe method is used to detect intracellular reactive oxygen species (ROS). The results of the flowmetry analysis showed (Figure 2B) that DIP pretreatment reduced NaAsO2-induced intracellular ROS levels in L-02 cells compared with the control.

The changes in cellular mitochondrial membrane potential were detected by JC-1 Mitochondrial Membrane Potential Assay Kit, and the results showed that the proportion of NaAsO_2_-induced elevated mitochondrial membrane potential depolarization was reduced in L-02 cells after DIP pretreatment (*p* < 0.05, Figure 2C). Meanwhile, DIP was shown to inhibit the protein expression of PINK1, Parkin, p62, and LC3 II/І by a Western blot assay (Figure 2D). These results suggest that DIP can inhibit mitophagy injury induced by NaAsO_2_ through the PINK1/Parkin pathway.

### 3.4. Effects of Scavenging ROS on NaAsO_2_-Induced Mitochondrial Structural Damage

**Figure 3:**
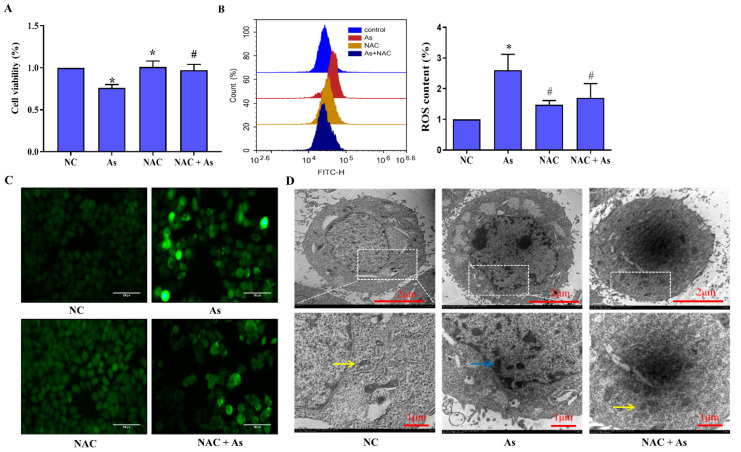
Effects of scavenging ROS on NaAsO2-induced mitochondrial structural damage. (**A**) CCK8 assay was used to detect cell viability of NAC pretreatment. (**B**) Flow cytometry detection of NAC inhibits the accumulation of ROS in L-02 cells induced by NaAsO_2_. (**C**) The ROS detection kit fluorescently detects the effect of NAC on the ROS in L-02 cells induced by NaAsO_2_. Scale bar = 100 μm. (**D**) Transmission electron microscopy was used to detect the effect of NaAsO_2_ on intracellular mitochondria and the effect of NAC. In the figure, the yellow arrow points to normal or slightly damaged mitochondria, and the blue arrow indicates the appearance of mitochondria with disappeared mitochondrial cristae, swelling, and enlargement. * *p* < 0.05 compared with the control group. # *p* < 0.05 compared with the As group.

To clarify the mechanism of DIP protecting L-02 cells from mitophagy injury, we pretreated L-02 cells exposed to NaAsO_2_ with ROS-specific scavenger NAC. The results of CCK8 showed that after NaAsO_2_ treatment, the cell survival rate was significantly (*p* < 0.05) reduced; after pretreatment with NAC, the inhibition of NaAsO_2_ cell survival rate was alleviated (Figure 3A). The result showed that NaAsO_2_ triggered ROS generation and NAC can effectively eliminate the generation and accumulation of ROS induced by NaAsO_2_.

As shown in Figure 3B,C, the results of the DHFH-DA fluorescent probe method showed that the intracellular ROS content of the As group was higher (*p* < 0.05) than that of the control group. After NAC pretreatment (*p* < 0.05), the intracellular ROS decreased significantly compared with the As group. Observation under a fluorescence microscope revealed that there were few green fluorescent cells in the control group and the NAC group. The green fluorescent cells in the As group increased while the green fluorescent cells decreased after NAC pretreatment. These results indicated that NaAsO_2_ can cause the accumulation of ROS in L-02 cells, while NAC obviously inhibits the accumulation of ROS in L-02 cells induced by NaAsO_2_.

As shown in Figure 3D, the results of the transmission electron microscopy showed that the mitochondrial cristae were complete and clear. After exposure to 10 μM NaAsO_2_, the mitochondria in the cells were swollen and deformed, the mitochondrial cristae were broken or had disappeared, a vacuole-like structure appeared, and mitochondrial autophagosomes wrapped in a double membrane were visible; the damage to the mitochondria in the NAC + As group was significantly (*p* < 0.05) alleviated compared to the As group.

### 3.5. Effects of ROS Clearance on Mitophagy-Related Genes and Proteins in the PINK1/Prkin Pathway

**Figure 4:**
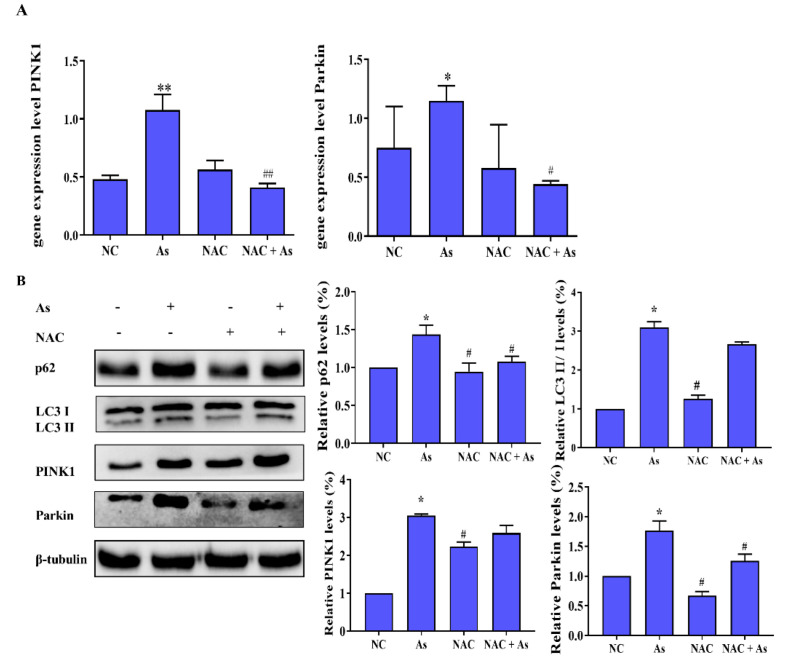
Effects of ROS clearance on mitophagy-related genes and proteins in PINK1/Prkin pathway. (**A**) RT-qPCR was used to detect the changes of PINK1 and Parkin mRNA in mitophagy induced by NaAsO2 treated with NAC. (**B**) Western blotting was used to detect the expression of mitophagy-related proteins induced by NaAsO2 after NAC pretreatment. * *p* < 0.05, ** *p* < 0.01 compared with the control group. # *p* < 0.05, ^##^ *p* < 0.01 compared with the As group.

Intracellular RNA was extracted for RT-QPCR analysis, as shown in Figure 4A,B. After NaAsO_2_ treatment, the mRNA expressions of PINK1 and Parkin increased significantly, and the differences were statistically significant (*p* < 0.05). However, NAC pretreatment reduced the expression of PINK1 and Parkin mRNA, suggesting that ROS plays an important role in the up-regulation of PINK1or Parkin mRNA by NaAsO_2_ in L-02 cells.

As shown in Figure 4B, Western blot results showed that the expression levels of p62, LC3 II/І, and PINK1 protein following exposure to the NaAsO_2_ increased (*p* < 0.05), and the expression of Parkin protein in the cytoplasm decreased (*p* < 0.05), indicating that NaAsO_2_ induced PINK1/Parkin-mediated mitophagy. In contrast, the expression levels of p62, LC3 II/І, PINK1, and Parkin protein were significantly (*p* < 0.05) reduced in the NAC+As group compared with the As group, indicating that ROS induced by NaAsO_2_ mediates the PINK1/Parkin pathway.

## 4. Discussion

Dictyophora is a valuable dual-use fungus, rich in vitamins, proteins, polysaccharides, and other active substances [19]. DIP is extracted from Dictyophora and consists of a variety of monosaccharides that have been shown to have anti-inflammatory and immunomodulatory properties [20]. It has great exploitation value in medicine and food. Kanwal et al., using the Illumina MiSeq platform, found that DIP decreased endotoxemia (via lipopolysaccharide) and pro-inflammatory cytokine (TNF-α, IL-6, and IL-1β) levels and increased the expression of tight junction related proteins (claudin-1, occludin, and zonula occludens-1). This demonstrates the effect of DIP in reducing inflammation and lowering endotoxin levels [21]. Wang et al. used DIP to intervene in C57BL/6 mice with dextran sodium sulfate-induced colitis and showed that DIP could regulate macrophage polarization and restore intestinal barrier function, suggesting that DIP could be used as a functional food or nutritional agent to improve intestinal inflammation [22].

Guizhou province in China is a typical karst landscape, and some of the soils in the region are rich in the element As [3]. We previously used Bayesian reference dosing to analyze the possible maximum acceptable cumulative arsenic exposure level for liver damage caused by arsenic from coal burning in Guizhou province. For liver damage caused by arsenic from coal burning, the recommended maximum acceptable cumulative arsenic exposure level is 7120 mg. With the increase of cumulative exposure, liver injury was aggravated, resulting in significant changes in serum ALT, AST, and other indicators [23]. Our previous study has confirmed the protective effect of DIP against liver injury caused by arsenic through in vivo experiments in rats, but the mechanism is unclear [14]. In addition, we found that arsenic exposure can cause mitophagy in human liver cells (L-02 cells), thereby mediating apoptosis, and the PINK1/Parkin pathway plays an important role [9]. This is consistent with most studies. Jiang et al. found that lipocalin mediated mitophagy via the activation of the SIRT1-PINK1 signaling pathway, which is closely related to oxidative stress, inflammation, apoptosis, and mitochondrial dysfunction induced by lung ischemia-reperfusion injury in type 2 diabetic rats [24]. In addition, a study confirmed that probiotic pretreatment mediated PINK/ Parkin-induced autophagy and eliminated damaged mitochondria, thereby reducing induced apoptosis [25]. All of the above suggest a close relationship between autophagy and cell damage. Mitochondria are important functional organs that provide energy during normal life activities. Damage to mitochondria may lead to a variety of human diseases, including cancer, cardiovascular disease, neurodegenerative disease, and liver disease [26]. When mitochondria are damaged by starvation, oxidative stress, inflammation, ROS, or hypoxia, they will initiate mitophagy to protect themselves [27,28]. Mitophagy is a kind of selective autophagy, which is used to selectively remove damaged mitochondria or misfolded proteins in the cell. It plays a vital role in maintaining cell homeostasis and mitochondrial quality control [29]. Therefore, based on our previous evidence that DIP has a protective effect against As-induced liver injury, and that As can induce mitophagy and trigger cytotoxicity through the PINK1/Parkin pathway, our present study explored the mechanism of DIP protection against As-induced liver injury from the perspective of mitophagy to further enrich the pharmacological mechanism of Dictyophora.

Our study showed that L-02 cell survival decreased after NaAsO_2_ treatment and that protein expression of p62 and LC3 II/І increased with increasing exposure concentration and time. LC3 and p62 are the main proteins involved in autophagy and are key indicators for detecting cellular autophagy, so it is suggested that NaAsO_2_ can induce autophagy. The PINK1/Parkin pathway is currently a pathway that has been studied in the mitophagy pathway [30]. PTEN-induced kinase 1 (PINK1) is a Ser/Thr kinase that is mainly located on the outer membrane of mitochondria. Parkin RBR E3 ubiquitin protein ligase (Parkin) is mainly located in the cytoplasm and plays a key role in mitochondrial motility and size [31,32]. In general, the small amount of PINK1 produced is easily degraded by the proteasome, so it cannot be detected or the amount detected is small; but when the membrane is depolarized or the mitochondrial complex is damaged, the PINK1 dimer is autophosphorylated, thereby activate kinase and bind to ubiquitinated Parkin. As PINK1 can quickly respond to changes in mitochondrial stressors, it can be used as a sensor of mitochondrial damage [33,34]. The results of this experiment showed that compared with the control group, the expression level of PINK1 protein increased in L-02 cells treated with NaAsO_2_, while the level of Parkin protein in the cytoplasm decreased, indicating that NaAsO_2_ induced PINK1/Parkin-mediated mitophagy.

DIP has antioxidant capacity. Liu et al. found that DIP exhibited the strong reducing ability and scavenging activity of DPPH, superoxide and hydroxyl radicals in vitro antioxidant assays, indicating the potential of DIP as a natural antioxidant resource [35]. Our previous study also found that DIP unregulated the expression of Bax and caspase-3 genes and reduced Bcl-2/Bax heterodimer formation, regulated the HCC-LM3 cell cycle, and promoted the role of the cellular mitochondrial apoptotic pathway, suggesting that DIP has potential therapeutic value for liver injury [16]. In the organism, one of the main sources of ROS is the substrate end of the respiratory chain in the inner mitochondrial membrane, and mitochondrial damage leads to an increase in ROS, triggering increased activity of DPPH and oxidative and hydroxyl radicals [36]. Thus, does DIP inhibit arsenic-induced mitophagy in the liver, and is the mechanism of DIP protection of the liver related to ROS?

We found that DIP had an inhibitory effect on NaAsO_2_-induced L-02 cytotoxicity by assaying the cellular activity after DIP pretreatment. Detection of intracellular ROS by DCFH-DA fluorescent probe assay revealed that NaAsO_2_ could induce intracellular ROS production, while DIP could scavenge excess ROS. It is known that increased ROS disrupts the mitochondrial membrane and alters the membrane permeability, reducing the concentration difference between ions inside and outside the membrane by free diffusion, which leads to a decrease in membrane potential [37]. Therefore, we used JC-1 probe staining to detect the proportion of mitochondrial membrane potential depolarization in cells and found that NaAsO_2_ induced an increase in the proportion of mitochondrial membrane potential depolarization, and this proportion decreased after DIP treatment. This shows that DIP has a protective effect on NaAsO_2_-induced mitochondrial damage; however, does DIP interfere with the mitochondria process?

We further examined the expression of p62, LC3 II/І, PINK1, and Parkin proteins and found that pretreatment with DIP reduced the expression of these proteins compared to NaAsO_2_ exposure alone. The p62 protein, also known as the SQSTM1 protein, is involved in autophagy as a linker protein connecting LC3 and ubiquitinated substrates [38]. As an important autophagic bridging protein, p62 is responsible for the degradation of ubiquitinated proteins into autophagosomes and is a marker of autophagic flow, and reduced levels of p62 imply that autophagic flow is protected. In summary, we hypothesize that during NaAsO_2_-induced mitochondrial injury. DIP may reduce the proportion of mitochondrial membrane potential depolarization by clearing excess ROS scavenging, activating the PINK1/Parkin pathway to regulate p62 and LC3 II/І protein expression, and alleviating arsenite-induced mitophagy injury.

Finally, we directly used a specific scavenger of ROS (N-Acetyl-L-cysteine. NAC) to verify our speculation, and the results were as we thought. Pretreatment of L-02 cells with NAC followed by arsenic exposure inhibited NaAsO_2_-induced mitophagy along with a decrease in intracellular ROS levels. LC3 II/І, p62 expression levels were also reduced. We further investigated the relationship between NaAsO_2_-induced production of ROS and mitophagy and the PINK1/Parkin pathway. The results showed that NaAsO_2_ treatment increased the expression level of PINK1 and Parkin, while NAC pretreatment alleviated this phenomenon. Moreover, we found by transmission electron microscopy results that more morphologically abnormal mitochondria were present in NaAsO_2_-treated cells compared with control cells, forming mitochondrial autophagosomes, while structural damage of mitochondria was significantly inhibited by NAC pretreatment. These results suggest that ROS scavenging plays an important role in the protective mechanism of DIP against NaAsO_2_-induced mitophagy in L-02 cells.

This study provides new evidence for the hepatoprotective mechanism of Dictyophora and provides a reference for the study of the pharmacological mechanism of Dictyophora polysaccharide. However, there are limitations, because the molecular mechanism of mitophagy is complex; among the many mechanisms of mitophagy, we only studied the PINK1/Parkin pathway. In addition, Dictyophora polysaccharide has a variety of beneficial effects on the human body and may not have a single reason for its hepatoprotective effect, so further research is needed on the hepatoprotective mechanism of Dictyophora polysaccharide.

## 5. Conclusions

**Figure 5:**
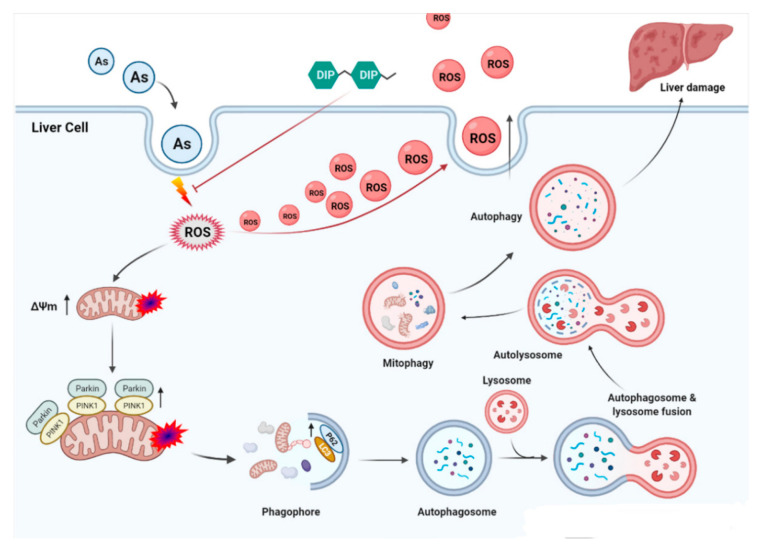
DIP attenuates PINK1/Parkin pathway-mediated mitophagy damage in L-02 cell through scavenging ROS. Mitophagy was found in hepatocytes induced by arsenic exposure, and DIP exerts a protective effect on hepatocyte by scavenging ROS, which could restrain arsenic-induced mitochondrial membrane potential depolarization and PINK1/Parkin pathway-mediated mitophagy to inhibit hepatocyte injury.

In summary, mitophagy was found in hepatocytes induced by arsenic exposure, and DIP exerts a protective effect on hepatocyte by scavenging ROS, which could restrain arsenic-induced mitochondrial membrane potential depolarization and PINK1/Parkin pathway-mediated mitophagy to inhibit hepatocyte injury (Figure 5).

## Data Availability

All data and materials are contained and described within the manuscript.
